# Fully Automatic Atrial Fibrosis Assessment Using a Multilabel Convolutional Neural Network

**DOI:** 10.1161/CIRCIMAGING.120.011512

**Published:** 2020-12-15

**Authors:** Orod Razeghi, Iain Sim, Caroline H. Roney, Rashed Karim, Henry Chubb, John Whitaker, Louisa O’Neill, Rahul Mukherjee, Matthew Wright, Mark O’Neill, Steven E. Williams, Steven Niederer

**Affiliations:** 1 Biomedical Engineering and Imaging Sciences, King’s College London, United Kingdom (O.R., I.S., C.H.R., R.K., H.C., J.W., L.O., R.M., M.O., S.E.W., S.N.).; 2 Cardiology Department, St. Thomas’ Hospital, London, United Kingdom (M.W., M.O.).

**Keywords:** atrial fibrillation, deep learning, fibrosis, magnetic resonance imaging

## Abstract

**Background:**

Pathological atrial fibrosis is a major contributor to sustained atrial fibrillation. Currently, late gadolinium enhancement (LGE) scans provide the only noninvasive estimate of atrial fibrosis. However, widespread adoption of atrial LGE has been hindered partly by nonstandardized image processing techniques, which can be operator and algorithm dependent. Minimal validation and limited access to transparent software platforms have also exacerbated the problem. This study aims to estimate atrial fibrosis from cardiac magnetic resonance scans using a reproducible operator-independent fully automatic open-source end-to-end pipeline.

**Methods:**

A multilabel convolutional neural network was designed to accurately delineate atrial structures including the blood pool, pulmonary veins, and mitral valve. The output from the network removed the operator dependent steps in a reproducible pipeline and allowed for automated estimation of atrial fibrosis from LGE-cardiac magnetic resonance scans. The pipeline results were compared against manual fibrosis burdens, calculated using published thresholds: image intensity ratio 0.97, image intensity ratio 1.61, and mean blood pool signal +3.3 SD.

**Results:**

We validated our methods on a large 3-dimensional LGE-cardiac magnetic resonance data set from 207 labeled scans. Automatic atrial segmentation achieved a 91% Dice score, compared with the mutual agreement of 85% in Dice seen in the interobserver analysis of operators. Intraclass correlation coefficients of the automatic pipeline with manually generated results were excellent and better than or equal to interobserver correlations for all 3 thresholds: 0.94 versus 0.88, 0.99 versus 0.99, 0.99 versus 0.96 for image intensity ratio 0.97, image intensity ratio 1.61, and +3.3 SD thresholds, respectively. Automatic analysis required 3 minutes per case on a standard workstation. The network and the analysis software are publicly available.

**Conclusions:**

Our pipeline provides a fully automatic estimation of fibrosis burden from LGE-cardiac magnetic resonance scans that is comparable to manual analysis. This removes one key source of variability in the measurement of atrial fibrosis.

## Abstract 2

Supplemental Digital Content is available in the text.

**CLINICAL PERSPECTIVE**

Atrial late gadolinium enhancement cardiac magnetic resonance scans provide an estimate of fibrosis burden and a wide range of clinical applications. Preablation scans, for instance, could be used to refine selection of patients for catheter ablation and to predict atrial fibrillation recurrence following intervention. The provided fibrosis characterization could conceivably inform personalized ablation strategy or enable patient-specific modeling for predicting atrial fibrillation drivers. It could also be used as a factor in stroke risk. Each of these applications, however, is heavily dependent on operator’s trust in a reproducible technique. Conventional late gadolinium enhancement cardiac magnetic resonance image analysis techniques are performed on proprietary software and have manual components that are subjective and susceptible to intraoperator and interoperator variation. We developed and validated a transparent, reproducible, and objective workflow for estimating atrial fibrosis from late gadolinium enhancement cardiac magnetic resonance fully automatically. Our workflow reduces operator dependent variability and the number of patients required to power clinical studies. The workflow is open-source to provide other centers with the ability to estimate atrial fibrosis, and hopefully contribute to its further development, as the low number of centers using atrial cardiac magnetic resonance imaging is a limitation for open research in this area.

Atrial fibrillation (AF) is the most common arrhythmia. Guidelines recognize that AF is complex for clinicians to manage.^1^ Patients can be treated pharmacologically, by catheter ablation to isolate or remove aberrant atrial tissue, or by atrioventricular node ablation coupled with pacemaker implantation. Unfortunately, pharmacological treatments have profound side effects, AF may recur in 50% of AF ablation cases, and pacemaker dependency has inherent risks. Each patient requires a treatment specific to their AF and selecting the optimal treatment for each patient remains a daily clinical challenge.^1^

Precision cardiology requires accurate characterization of the disease phenotype for each patient.^2^ Pathological atrial fibrosis is a major contributor to sustaining AF and has been proposed as a biomarker to guide personalized treatment.^3^ Late gadolinium enhancement (LGE) cardiac magnetic resonance imaging (CMR) has been proposed as a method for estimating atrial fibrosis and the fibrosis burden estimated by LGE-CMR correlates with patient response to AF ablation. However, widespread adoption of LGR-CMR for informing clinical decisions has remained controversial. This is partly due to a lack of consensus on how best to process images, as well as unreliable image quality of scans, with only 17% to 40% of images being diagnostic at leading centers.^4^ Minimal validation and limited access to open-source software platforms have also exacerbated the problem.^5^

We recently proposed a pipeline for the reproducible processing of LGE-CMR scans.^6^ However, the pipeline still required the manual segmentation of the left atria (LA) and labeling of important anatomic structures, including the pulmonary veins (PV), mitral valve (MV), and left atrial appendage (LAA). This segmentation process is time consuming (15 minutes per case on average), requires significant expertise, and is prone to interobserver variability. Motivated by these factors, several prior studies have benchmarked automatic atrial segmentation methods^7,8^ but most previous attempts were single label methods, which focused on delineating the LA blood pool including its surrounding anatomies and not on measuring LGE fibrosis burden.^9–12^

PVs and MV have different tissue characteristics to the LA body and are more fibrotic.^13^ LAA is a trabeculated structure with a variable wall thickness,^14^ which may bias the fibrosis analysis. Furthermore, there is a large variability in the morphology between patients. It is, therefore, desirable to restrict the assessment to the body of the LA. A multilabel segmentation approach can alleviate these issues.

In this article, we propose a novel end-to-end automatic pipeline for estimation of atrial fibrosis from LGE-CMR scans, including a convolutional neural network (CNN) for blood pool, PVs, LAA, and MV segmentation. We validated our methods on a large 3-dimensional (3D) CMR data set from 207 manually annotated scans. Furthermore, our pipeline was implemented as part of a user-friendly platform that provides validated, transparent, and reproducible estimation of atrial fibrosis from LGE-CMR in <3 minutes. This was achieved through a deep learning approach with potential applications in the diagnosis and stratification of patients with AF.

## Methods

**Figure 1.**
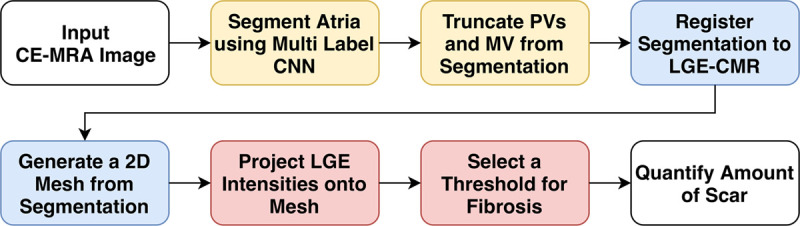
**Fibrosis estimation pipeline.** A fully automatic pipeline for estimation of atrial fibrosis from cardiac magnetic resonance (CMR) scans. A convolutional neural network (CNN) makes segmentation of the left atria (LA) possible without user intervention. Deep learning components are in yellow. Blue represents conventional image processing techniques and red illustrates assessment of fibrosis using late gadolinium enhancement (LGE)-CMR. Quality control assessment is performed after every step in the pipeline. 2D indicates two dimensional, CE-MRA, contrast enhanced magnetic resonance angiogram; MV, mitral valve; and PV, pulmonary vein.

Here, we first introduce the clinical data used, followed by the network proposed to segment LA structures. We then conclude with the sequence of processes used to automate analysis of fibrosis. Figure 1 displays the components of this pipeline. All the codes from this section have been made publicly available at the CemrgApp repository and can be accessed at http://www.cemrgapp.com.

### Clinical Data

CMR imaging was performed on 1.5 T MR scanners (Ingenia, Philips Healthcare, Best, Netherlands and Magentom Aera, Siemens Leipzig, Germany). All patients underwent detailed assessment including LA volumes and function and 3D LGE assessment of LA fibrosis. An ECG-triggered, contrast enhanced magnetic resonance angiogram (CE-MRA) 3D data set was acquired to delineate the LA endocardial border 90 seconds after gadolinium 0.2 mmol/kg (Gadovist, Bayer HealthCare Pharmaceuticals, Berlin, Germany) administration at a rate of 0.3 mL/s. LGE images were acquired using a 3D, inversion recovery, spoiled gradient echo acquisition, which was performed 20 minutes after administration of gadolinium with coverage to include the whole of the LA in axial orientation. Respiratory gating artefact was minimized by positioning the respiratory navigator as laterally as possible over the right posterior aspect of the diaphragm. The inversion time was determined from an inversion time mapping sequence performed immediately before the LGE acquisition to ensure adequate nulling of the ventricular myocardium. Control patients were recruited at the time of attendance to the rapid access chest pain clinic, and written consent was acquired, and ethical approval was granted by the Health Research Authority (18/LO/1803). Preablation and postablation patients underwent CMR imaging on clinical grounds. Ethical approval was granted for the retrospective analysis of this anonymized data without explicit written patient consent (18/HRA/0083).

Five clinicians with experience in CMR manually analyzed 207 of these atrial scans, where one of them processed 147 scans and the other four the remaining 60 scans twice to test the interobserver reproducibility of the pipeline. The 60 scans included 20 preablation, 20 postablation, and a further 20 non-AF control scans. Sequential patients documented to be in sinus rhythm at the time of the scan were chosen for inclusion. Each of these 4 clinicians processed 10 scans from each category. All scans in our data set are from different patients. The data set scans contained between 64 and 150 slices. The individual 2-dimensional (2D) slices were between 250 and 450 by 223 to 403 pixels. The resolution was between 0.92 and 1.3 by 0.92 to 1.3 by 2 mm. As the in-plane scan size varied, each 2D slice was resampled by rescaling to 1 by 1 mm isotropic resolution, with 320 by 320 pixels. Intensities of 3D CE-MRA were also rescaled using minimum to maximum normalization to range between 0 and 1. The normalization makes the training of CNN robust in dealing with variable image contrasts.

### Atrial Labels Generation

To develop a CNN for segmenting the LA and its surrounding structures, we first generated manually labeled data, detailed as follows.

#### Blood Pool Labels

A 3D segmentation of the LA endocardium was constructed using a region growing tool to define the blood pool from a CE-MRA scan. The LA has a very thin myocardial wall making it difficult to image even at the best resolutions available. CE-MRA scans allow confident identification of the endocardial surface. These manual labels provided the training samples for our automatic CNN approach of segmenting the blood pool.

#### Ostia Localization and Vein Labels

**Figure 2.**
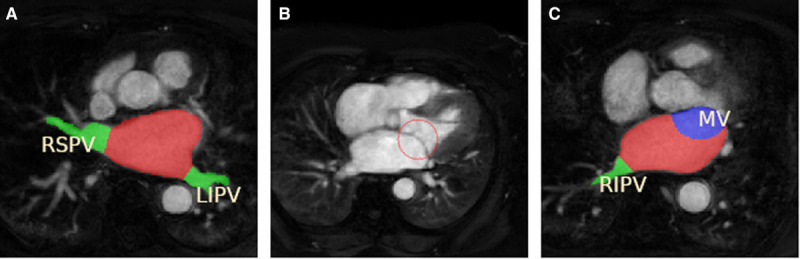
**Atrial labels generation.** **A**, Blood pool and pulmonary vein labels are generated to provide learning data for our multilabel convolutional neural network. **B**, The sphere used to remove mitral valve (MV) is illustrated in red. **C**, The MV label is shown in blue, together with the pulmonary veins label in green and left atrium body blood pool in red. LIPV indicates left inferior pulmonary vein; RIPV, right inferior pulmonary vein; and RSPV, right superior pulmonary vein.

To prepare training samples for other anatomic labels, a reproducible tool to remove the PVs, LAA, and MV from the blood pool segmentation was required. However, due to lack of clear anatomic landmarks defining the boundary between the LA body and these structures was not trivial. We made use of a Voronoi diagram^15^ to localize these boundaries. Further details about localization algorithm can be found in the Data Supplement. Throughout our work, we used a single label for the PVs and LAA. These labels, as seen in Figure 2A, were used in preparing training samples for our CNN to automatically identify the veins and eradicate the need of user guided clippers.

#### MV Labels

To accurately delineate the MV, the 3D image was manipulated in a multiplanar reconstruction viewer. The view was aligned so that the typical 2-chamber and 4-chamber views were seen, and the MV was visible en-face at the level of the orifice. Three points were then placed on the border of the orifice. A sphere generated by these 3 points could then be viewed to ensure that the sphere intersects with the valve tissue, but the atrial wall was spared. The 3 points could be manipulated to change the size and location of the sphere to ensure optimal placement. The intersection of the sphere and blood pool labeled the MV tissue and was used in the training of our CNN model. The sphere and labeled atria can be seen in Figure 2B and 2C.

### Multilabel CNN

**Figure 3.**
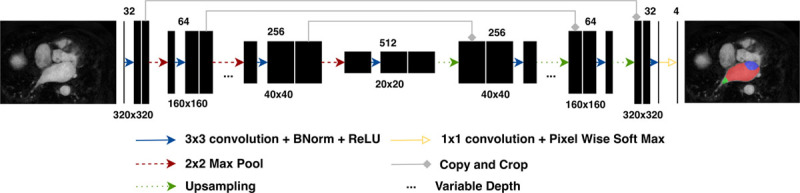
**Convolutional neural network.** General network architecture with 5 concatenations between contracting and expanding paths. The number of concatenations and number of feature maps in the output of the first convolution were hyperparameters during model selection (Data Supplement).

We developed our CNN in TensorFlow (https://www.tensorflow.org). The network used is a specialization of U-Net proposed by.^16^ The input of our convolutional network is a 320×320 resampled CMR image and the labels are hot-encoded with 3 channels for blood pool, PVs, and MV. The output is a probability map of the same size as the input for each label. The architecture of the network can be seen in Figure 3. Full details of the network architecture and its parameters can be found in the Data Supplement.

#### Network Training

The 207 3D CE-MRA data set was processed to generate 20 723 2D slices. Data augmentation techniques were also used to artificially increase the number of 2D slices to 1 068 427. The augmented data was used for training and not the evaluation of the network. Effective training requires a balanced set of all labels in the training data. Preliminary attempts at direct training on the entire 2D slices was ineffective as the PVs and MV pixels appeared in a subset of slices, and labels were sparse and unbalanced. To alleviate this, slices which contained at least 50 pixels for each of the blood pool, PVs, and MV labels were extracted from the training set. These were used to train the model initially before introducing the rest of the slices. This was done to prevent the network from receiving too many slices without the LA labels, which could potentially lead to an under segmentation of the anatomy. The 207 patients (not the 2D slices) were randomly split into a conventional 70% training, 10% validation, and 20% testing sets. Full details of the data augmentation and training techniques are available in the Data Supplement.

#### Evaluation

The network provides a probabilistic estimate of the labels for each pixel. A threshold at 50% of estimated probabilities was applied to restrict the pixels to binary values for the labels. Unconnected labeled islands were removed automatically from the reconstructed 3D images by keeping only the largest connected tissue as the final LA segmentation. The labels generated by the network were then evaluated against the manually labeled data. To avoid potential biases introduced in different training sessions, all results are based on a 3-time repeated random sub-sampling cross validation method.

### Fibrosis Estimation

The output from the segmentation network was used to automate the estimation of fibrosis by applying the following image processing steps from the pipeline. Once a segmentation is obtained from CE-MRA images, the fibrosis content is estimated by analyzing the maximum transmural image intensities from LGE-CMR scans.

#### Processing the Segmentation

The output labels from the network allowed us to isolate the body of atria and remove PVs and MV from the scans by setting the unwanted regions to a background value. Rigid body registration was then used to align the CE-MRA and LGE-CMR scans. Matching of the 2 images was performed by finding the rotations and translations that optimized the normalized mutual information as the similarity function of the 2 images. The displacement field obtained from the registration of 2 images was used to transform the segmentation image created from the network to the LGE-CMR space. An endocardial surface model was finally created from the segmentation using the marching cubes algorithm. We used the Medical Image Registration ToolKit (https://mirtk.github.io) for these steps. MIRTK’s full registration parameters set can be found in the Data Supplement.

#### Detection of Fibrosis

Normals were taken, 3 mm externally and 1 mm internally, to the nodes of the surface mesh, and a maximum intensity projection technique was used to interrogate the LGE-CMR image. Three millimeter was chosen externally to align with approximate atrial wall thickness^17^ and the small, 1 mm, internal projection allowed a degree of registration error without incorporating respiratory navigator artifact. An assumption of 3 mm is on the thicker side of atrial wall thickness measurements.^18^ We, therefore, examined the effects of varying normal projections length on the estimated global fibrosis score to make sure that atrial fat did not significantly alter the results.

A 3-voxel size erosion of the LA blood pool was used to generate a blood pool segmentation from which the mean blood pool signal intensity and SD were calculated for use as a reference value for atrial wall assessment. Global fibrosis burdens were calculated using previously published thresholds: image intensity ratio (IIR) of 0.97, IIR of 1.61,^19^ and mean blood pool signal +3.3 SD.^20^

### Statistics

The Shapiro-Wilk test with a significance level of 0.05 was used to determine if patient demographic data were normally distributed. Normally distributed variables are presented as mean±SD, and non-normally distributed variables as median interquartile range.

Segmentation results were evaluated using the Dice coefficient, accuracy, sensitivity, specificity, and precision. The difference between automatically and manually calculated atrial surface area, volume, and sphericity were evaluated by the mean absolute and relative errors of the measurements.

The predefined fibrosis thresholds were applied at a voxel level for each patient, and the percentage of surface elements that were above the threshold were reported, resulting in a continuous fibrosis burden variable between 0% to 100%. Automatic versus manual fibrosis estimations were compared with the intraclass correlation coefficients (ICC), using a one-way random-effects model of interobserver reproducibility measurements with absolute agreement. ICC of 0.41 to 0.60 was interpreted to represent moderate, 0.61 to 0.80 good, and above 0.80 as excellent agreement. The Pearson correlation coefficient between automatic and manual fibrosis scores and the root mean square error between the 2 scores were also computed.

The association of automatic fibrosis burdens estimates with arrhythmia recurrence from a cohort of 89 patients were analyzed by binomial logistic regression, and the area under the curve values of receiver operator characteristic curves were reported. The fibrosis scores associated the recurrence and no recurrence groups were compared using a Student *t* test with a significance level of 0.05.

Multiple comparisons between groups with variable wall thickness were made by a one-way ANOVA and with a significance level of 0.05. All statistical analyses were performed in MATLAB (Version: R2018a, Natick, Massachusetts: The MathWorks Inc).

## Results

In this section, we first evaluate the accuracy of our optimized CNN in segmenting LA structures. We then compare the reproducibility of the CNN segmentations compared with interobserver variability. Finally, we measure the accuracy and reproducibility of automatically calculated fibrosis burdens. The patient demographic characteristics are summarized in Table 1. The samples in our work are based on real world CMR scans acquired for routine patient care, hence a wide range of AF duration is present in the data set.

**Table 1.**
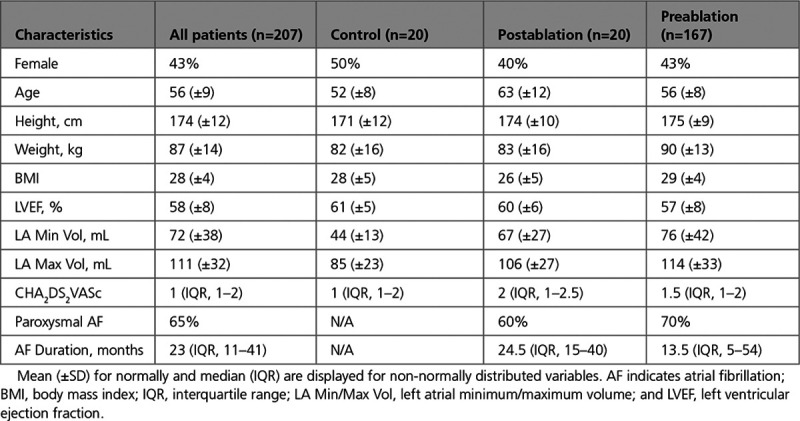
Patient Demographics

### Automatic Segmentation Results

Following hyperparameter optimization (Table II in the Data Supplement), we evaluated the optimal network model using Dice, accuracy, sensitivity, specificity, and precision measurements for each of the blood pool, PVs, and MV labels. Table 2 summarizes these metrics. Table 3 lists the average manual and model predicted measurements for the LA surface area, volume, and sphericity. The mean absolute and relative errors of the measurements are also displayed for comparison.

**Table 2.**
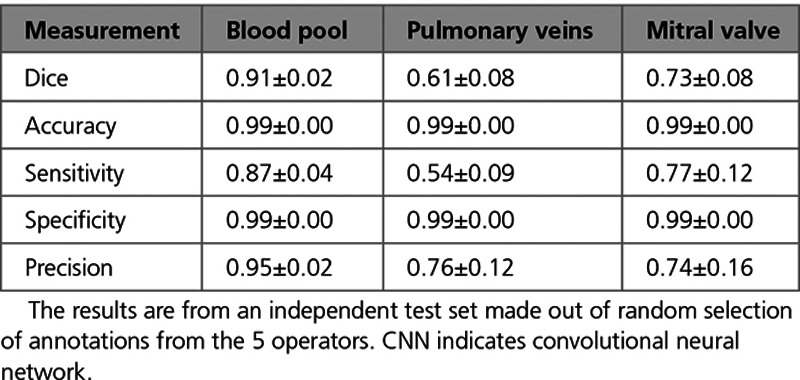
Average Segmentation Results Obtained From the CNN Network

**Table 3.**
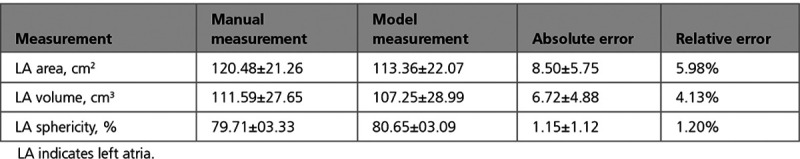
Comparison of Mean LA Area, Maximum Volume, and Sphericity Measurements Obtained From the Network and Manual Annotations on the Test Data Set

#### Comparison of Automatic Results to Interobserver Reproducibility

**Figure 4.**
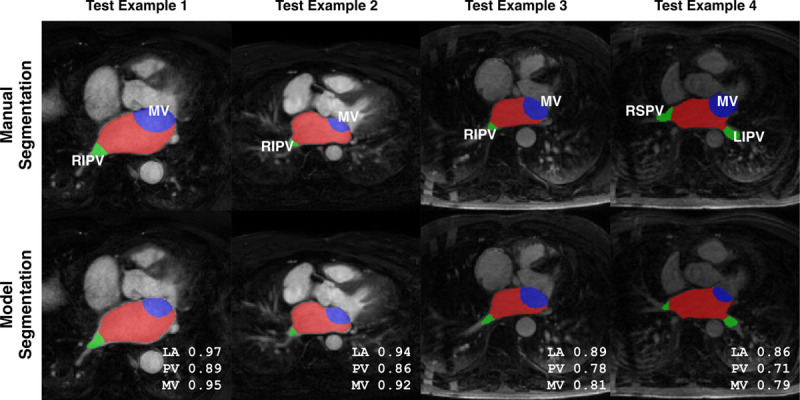
**Automatic and manual delineations.** The middle slice in each test image is shown, and images are ordered according to descending Dice scores from left to right. Blood pool is in red, mitral valve is in blue, and veins are shown in green. LA indicates left atrium; LIPV, left inferior pulmonary vein; MV, mitral valve; PV, pulmonary vein; RIPV, right inferior pulmonary vein; and RSPV, right superior pulmonary vein.

Sixty out of 207 of the scans were analyzed twice by 2 independent trained operators to analyze interobserver reproducibility. The mutual agreement of operators assessed using the Dice score for blood pool, PVs (including LAA), and MV labels were 0.85, 0.40, and 0.65, respectively. The CNN outperformed the interobserver reproducibility results for all anatomic labels. Due to multiple operators annotating different scans, we evaluated the effect of training and validating the network on data sets generated by different operators in the Data Supplement. Figure 4 illustrates examples of delineated boundaries from network prediction compared with their manually annotated boundaries.

### Automatic Fibrosis Estimation Results

**Figure 5.**
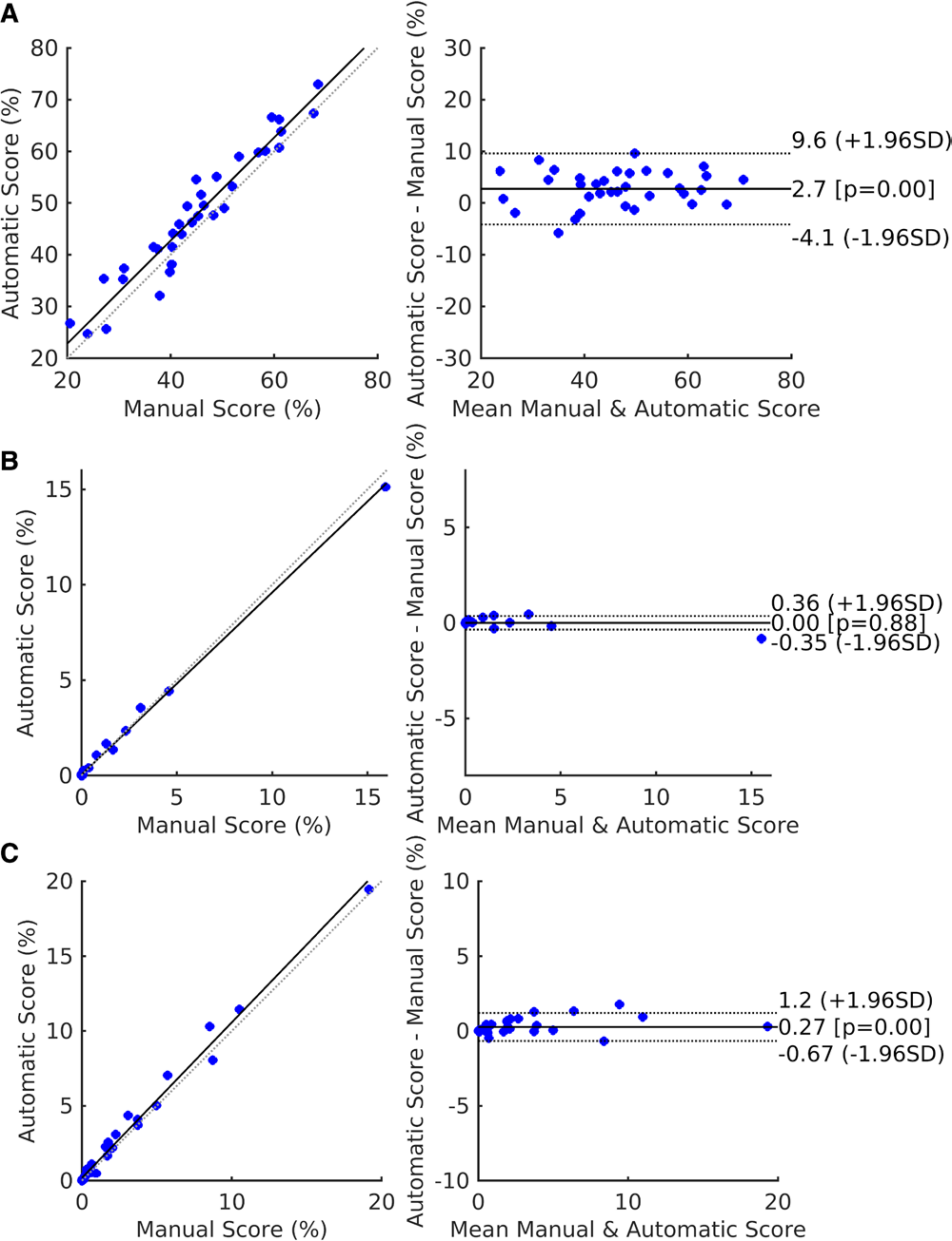
**Fibrosis scores plots.** Scatter and Bland-Altman plots from automatic and manual fibrosis predictions are shown when using (**A**) image intensity ratio (IIR) 0.97, (**B**) IIR 1.61, and (**C**) mean+3.3 SD thresholds. Using the IIR 1.61 threshold results in a large number of points clustering around zero. Results are from the test set.

Automatic versus manual fibrosis estimation results were evaluated using the ICC. The correlation with manually generated results using all 3 thresholds (IIR, 0.97; IIR, 1.61; and mean blood pool, +3.3 SD) were excellent. Table 4 summarizes all 3 thresholds tested using ICC as well as other correlation measurements. Figure 5 displays the scatter and Bland-Altman plots for the fibrosis scores using 3 different thresholds.

**Table 4.**
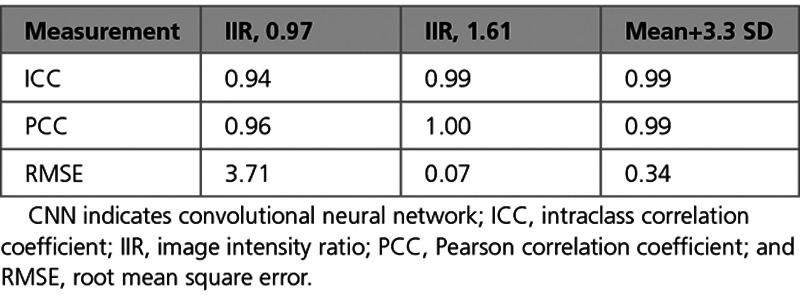
Fibrosis Scores Calculated From Segmentations Generated Manually by 5 Operators and Automatically by Our CNN

#### Comparison of Automatic Results to Interobserver Reproducibility

**Figure 6.**
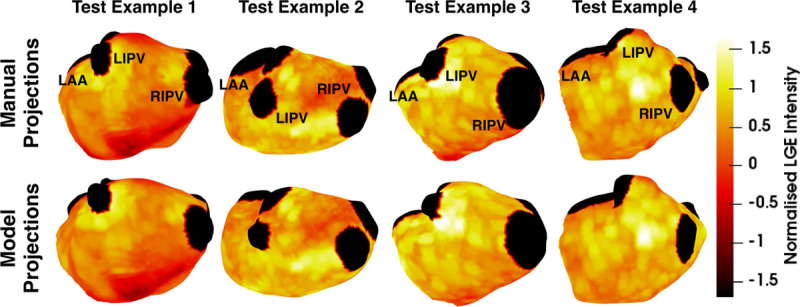
**Automatic and manual projections.** Examples of atrial meshes from the test set with fibrosis projected on their surfaces. Late gadolinium enhancement (LGE) intensities were normalized using the mean blood pool intensity. The pulmonary veins are clipped and represented in black. More fibrotic regions are depicted in yellow. LAA indicates left atrial appendage; LIPV, left inferior pulmonary vein; and RIPV, right inferior pulmonary vein.

Comparing the automatically obtained fibrosis score, ICC values to the interobserver ICC for the manual repeat segmentation data set shows the automatic method performed better than the interobserver scores for 2 of the thresholds: 0.94 (CI, 0.93–0.97) versus 0.88 (CI, 0.80–0.93) and 0.99 (CI, 0.97–0.99) versus 0.96 (CI, 0.90–0.97) for IIR 0.97 and +3.3 SD thresholds, respectively. The IIR 1.61 threshold yields fibrosis scores close to zero: 0.99 (CI, 0.99–0.99) versus 0.99 (CI, 0.98–0.99). We report further details about examined permutations of training and testing sets based on different combinations of operators to test the robustness and fairness of fibrosis scores in the Data Supplement. Figure 6 displays examples of atrial meshes from test sets with their associated LGE-CMR intensities projected on the surface. Results derived from both manual and automatic segmentations are presented.

### Internal and Public Data Sets Validation Results

To confirm that automated fibrosis measurement can make clinically meaningful predictions, we applied our method to a previously reported data set of 89 preprocedure LGE-CMR scans and paired outcomes.^21^ Using preablation LGE-CMR images from patients receiving their first AF ablation, we tested if each of the 3 measures of fibrosis burden, considered here, were higher in patients whose AF recurred within 12 months of the procedure. Mean fibrosis burden was significantly higher in the recurrence group, when measured using IIR, 0.97 (32.3 versus 47.8; *P*=0.003; area under curve (AUC)=0.692) and +3.3 SD (4.6 versus 7.6; *P*=0.03; AUC=0.631) thresholds. However, the IIR 1.61 threshold, which is used to measure dense scar, did not result in a significant difference (4.4 versus 7.3; *P*=0.08; AUC=0.594) between the 2 groups, consistent with these patients not having received a prior ablation. We also validated our segmentation network against the 2013 and 2018 Atrial Segmentation Challenge data sets, achieving LA Dice scores of 0.90 and 0.89, respectively. Complete details are available in the Data Supplement.

## Discussion

Atrial fibrosis plays a central role in the AF substrate, having been identified histologically in patients with AF^22^ and patients with risk factors for AF.^23,24^ Despite this recognized role in AF, translating these key observations into clinical-applicable investigations or treatment pathways remains challenging.

Atrial LGE-CMR scans provide an estimate of fibrosis burden and a wide range of clinical applications. We, therefore, developed an open-source end-to-end automatic pipeline to facilitate the wider adoption of atrial LGE-CMR for estimating fibrosis measurements in research and clinical practice. Automatic fibrosis estimation from preablation scans, for instance, could be used to refine selection of patients for catheter ablation and to predict AF recurrence following intervention.^25,26^ The provided fibrosis characterization could conceivably inform personalized ablation strategy^27^ or enable patient-specific modeling for predicting AF drivers.^28^ It could also be used as a factor in stroke risk.^29^ Given the broad clinical uses of atrial LGE-CMR, there are potential advantages of widespread access to an automatic pipeline, as each of these applications is heavily dependent on the operator’s trust in a reproducible technique and the low number of centers using atrial CMR imaging is a limitation for open research in the area.

We extensively validated our pipeline and confirmed our network’s ability to segment the atrial anatomy against internal and public data sets. We showed that our automated method replicates manual assessment of fibrosis burden from LGE-CMR and demonstrated that the fibrosis measurements are significantly different in patients, who suffer AF recurrence following ablation. However, all methods for measuring fibrosis from LGE-CMR are limited by the lack of a generally accepted clinical groundtruth. Voltage mapping provides an assessment of structural remodeling in the atria but can be influenced by activation rate and direction,^30–32^ which may limit its current capacity to provide a reference groundtruth for fibrosis. The use of automated pipeline, described here, removes a key subjective component from LGE-CMR image analysis. This reduces variability and ensures reproducibility, which could facilitate its use for fibrosis burden estimates in clinical applications. For these reasons, we evaluated our automatic pipeline against an estimate of fibrosis determined manually by expert analysis of the scans.

In enabling other centers to deploy, and hopefully contribute to further development of our approach, a substantial barrier to atrial fibrosis imaging may be removed through use of our pipeline. Previous studies by us^6^ and others^4,5,33^ have described methods for measuring LGE-CMR atrial fibrosis burden. However, these methods have required manual steps performed by expert operators, which are invariably subject to intraobserver and interobserver errors. Here, we developed and validated a fully automated pipeline, which allows independent operators at independent centers to achieve the same fibrosis burden analysis, when analyzing an equivalent scan, reducing interobserver dependent variability. The pipeline also reduces the need for expert operators and the average processing time of a data set from 15 to 3 minutes.

The former fibrosis scoring systems separated patients into groups with bands of burden as small as 5%^34^ or reported a cutoff of 10% for predicting AF recurrence.^35^ To place patients within bins of this size, a precise measurement of fibrosis is required, as differences of 2% to 3% could significantly decrease the accuracy of the classification. We have previously shown that manual segmentation of the fibrotic PV, LAA, and MV is variable.^6^ Mislabeling these highly fibrotic regions can cause a meaningful error in the fibrosis burden. Previous automated atrial segmentation algorithms used a single label approach,^9–12^ so were unable to identify the PV and MV. By using a multilabel network, we were able to both automate the segmentation of the atrial body and more importantly label the LAA, PV, and MV of the atria, ensuring a consistent and reproducible measure of fibrosis estimate across all cases. As changes in image acquisition parameters may affect robustness of our method, we additionally tested the pipeline on the Atrial Segmentation Challenge data sets. Details of these tests are available in the Data Supplement.

### Limitations

Many groups only acquire an LGE-CMR scan. The proposed pipeline requires both CE-MRA and LGE-CMR. This may increase the time required for the scan. While our approach shows that it is possible to have reproducible image analysis techniques, reproducible images are still required for atrial fibrosis measurements to be used for clinical decisions. In our study, the inversion time was determined from an inversion time mapping sequence performed immediately before the LGE-CMR acquisition to ensure adequate nulling of the ventricular myocardium and only images of diagnostic quality were included for analysis.

Previous studies have shown that atrial wall thickness ranges from 1.1 to 6.5 mm in the posterior LA^14,36^ and, therefore, a reliable fibrosis assessment requires an accurate segmentation of the endocardial and epicardial wall. As fibrosis is presented after maximal intensity projection in our approach, information on the transmural distribution may be lost due to the variation in the thickness or failure of the segmentation network. We examined the effects of varying normal projections length from 1 to 6 mm to interrogate the LGE-CMR image. The results of a one-way ANOVA revealed no significant difference (*P*=0.06) in the fibrosis burdens calculated by considering the significance level as 0.05.

### Conclusions

This work combines the strength of deep learning with conventional image processing techniques to improve the speed and reproducibility of fibrosis estimation from LGE-CMR. This addresses one of the crucial steps in developing reproducible CMR atrial fibrosis quantification, which is a prerequisite for its wider adoption as a noninvasive assessment tool in informing patient care.

## Sources of Funding

Dr Niederer acknowledges support from the UK Engineering and Physical Sciences Research Council (EP/M012492/1, NS/A000049/1, and EP/P01268X/1), the British Heart Foundation (PG/15/91/31812, PG/13/37/30280), National Institutes of Health (NIH R01-HL152256), European Research Council (ERC PREDICT-HF 864055), and Kings Health Partners London National Institute for Health Research (NIHR) Biomedical Research Centre. Dr Williams acknowledges support from BHF funding (PG/19/44/34368 and FS/20/26/34952). Dr Roney acknowledges a Medical Research Council Skills Development Fellowship (MR/S015086/1). The Titan V GPUs used for this research were donated by the NVIDIA Corporation, Santa Clara, California.

## Disclosures

Dr Williams has a consulting agreement with Imricor Medical Systems.

## Supplementary Material


