# Supplementary material for: Fully Automatic Atrial Fibrosis Assessment Using a Multilabel Convolutional Neural Network
Source: Circ Cardiovasc Imaging. 2020 Dec 15;13(12):e011512. doi: 10.1161/CIRCIMAGING.120.011512 (PMC7771635; doi:10.1161/CIRCIMAGING.120.011512)
Supplement: Supplementary file 1 [file hci-13-e011512-s001.pdf]

SUPPLEMENTAL MATERIAL

Ostia Localisation and Truncation Methods

To isolate the body of LA, we developed automatic ostia localisation and PV truncation tools using a Voronoi diagram (1). This diagram was extracted from a surface mesh made from the blood pool segmentation. Each of the PVs was initially identified by placing landmarks in the centre of gravity for PV labels provided by the segmentation network. These landmarks represented the distal ends of the PVs in the mesh. Centrelines were then automatically drawn from these points to the centre of the atrial body, running through the veins. As the centrelines enter the body, the maximum area of the surrounding structure increases significantly. This inflection was used to identify the ostium. The rate of change of the area, shown in black polygons in Figure S1(a), was calculated as:

$$\Delta A_i = |A_i - A_{i-1}|, \tag{1}$$

where A_i is the area of a polygon at step i on the centreline. The possibility of finding a PV ostium was then determined based on the following conditions:

$$Ostium(i) = \begin{cases} False & \text{if } \Delta A_i \leq \Theta_{min} \\ \chi_B(\Delta A_i) & \text{if } \Theta_{max} \geq \Delta A_i \geq \Theta_{min} \\ True & \text{if } \Delta A_i \geq \Theta_{max} \end{cases}, \tag{2}$$

where Θ_{max} and Θ_{min} are predefined thresholds based on the average area of atria. To circumvent incorrect ostia localisation due to irregularities in the surface mesh, an additional function $\chi_B(\Delta A_i)$ was included that evaluated to *True* if the area increased monotonically at the identified ostia.

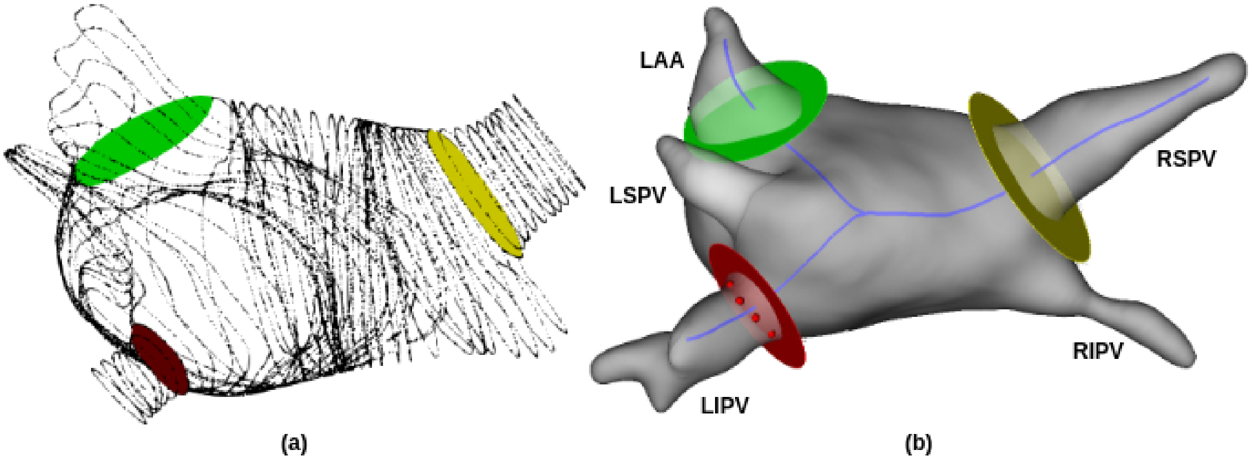

Figure S1: Ostia Localisation and Truncation: (a) A Voronoi diagram of the left atrium displaying partitioning of the geometry into polygons with area of surrounding structure encoded. (b) Computed centrelines are illustrated in blue running from PV landmarks to the centre of gravity. The localisation of ostia was achieved by analysing the change in the area of the surrounding structure. The colourful disks represent different PV truncation techniques. Yellow disks represent a fully automatic truncation method. Green disks represent the semi-automatic method, where the user has the option to define the size and angle of the clipper's shape. The red disk and its surrounding red points are the representative of a fully manual method, in which the user places a number of seeds around the PV to define the truncation path.

The PVs arrangements differ significantly between patients, which limit the use of the truncation approach described by Tobon-Gomez et al. (2). Their clipping is performed with an infinite plane, rendering it susceptible to unwanted cropping. To tackle this issue, we engineered a fully automatic clipper, which computed geometric properties of the PVs inner walls. This information was used to construct dynamically

shaped clippers. The clipper shapes helped to truncate the blood pool at the ostia and the isolated PVs were used to relabel the original segmentation.

In addition to the fully automatic clipper, we also devised two other types of truncation methods with different levels of manual interventions to provide flexibility. The semi-automatic type of truncation method exploited the visualization toolkit (VTK) implicit functions. The intersection of an infinite plane in conjunction with a user defined sphere created a ring shaped geometry, which resulted in a convex and flexible shape for the truncation of veins. For the manual method, the user picked a number of seeds on the surface mesh to define a contour. These seeds generated a custom shaped surface, which was then used for truncation of the veins. An example of this manual method is shown as a red disk in Figure S1(b). The wide variation in patient anatomy makes truncation tools such as these essential.

The labelled MV provided by the network undergoes three sequential steps to be automatically truncated from the atrial shell. First, the segmented MV is dilated and then converted to a surface using the MIRTK marching cubes algorithm. The surface of the MV is converted to an implicit function using the VTK Implicit PolyData Distance class. Finally, the implicit function is used to truncate the atrial shell using the VTK Clip PolyData filter, which allows for smooth clipped borders suitable for the rest of the estimation pipeline.

Convolutional Neural Network Implementation

We have a dedicated website to our open-source platform: http://www.cemrgapp.com. This website now has links to documentations, training videos, wiki pages, binary executables, and the public GitHub repository (https://github.com/CemrgAppDevelopers/CemrgApp), which host all the platform's source code. The convolutional neural network's source code used in this study is publicly available from this repository: https://github.com/OrodRazeghi/CemrgNet.

Network Architecture

The number of feature maps k in the convolutions of the first convolutional group was optimised as a hyperparameter during model selection. These 3 x 3 convolutional filters were connected to each other by 2 x 2 max pooling layers in the contracting path and up-convolutions in the expanding path. The number of max pooling layers, which is equal to the number of up-convolutions, was generalised to the depth of the network m as a hyperparameter. The number of feature maps in the output of the convolutions doubled after every max pooling layer, and this number halved after every up-convolution. The convolutions in these convolutional groups and those in the up-convolutions used padding, such that the output of the convolution was the same size as the input of this convolution. The convolutions in the convolutional blocks were followed by rectified linear unit (ReLU) activation and batch normalisation. The ReLU activation function was used for all layers apart from the last layer, which used a softmax activation function. The probabilistic output of this layer was considered to be the output of the model, which was thresholded at 50%.

```
# Network's Layers Parameters
Size of the convolution filter = 3 x 3
Size of the max pooling operation = 2 x 2
Output layer threshold = 0.5
```

Network Training

The types of augmentation used for training the network were rotation between -20 and +20, scaling between -20% and +20%, shearing between -10% and +10%, additive Gaussian noise with a mean of 0 and a standard deviation between 1 and 15 pixels, and contrast changing through the power law transformation. The proportion of the training set used for augmentation was tuned as to introduce a sufficient amount of new data but not cause overfitting by examining training and validation sets errors.

```
# Augmentation Parameters
Maximum rotation = +20
```

Minimum rotation = -20

Maximum scaling = +20

Minimum scaling = -20

Maximum shear = +10

Minimum shear = -10

Gaussian noise mean = 0

Gaussian noise minimum SD = 1

Gaussian noise maximum SD = 15

Transformation minimum gamma = 0.9

Transformation maximum gamma = 1.5

The adaptive moment estimation (ADAM) optimizer was used for optimisation of the network. An initial learning rate of 0.001 was selected for the ADAM optimizer and the exponential decay rates for the 1st and 2nd moment estimates were set to 0.9 and 0.999, respectively. The Dice coefficient was used as the cost function, since it was previously used in the 2013 Left Atrial Segmentation Challenge benchmark (2) and is better suited for datasets with a large label imbalance (3):

$$DICE = \frac{2|Label_{predicted} \cap Label_{groundtruth}|}{|Label_{predicted}| + |Label_{groundtruth}|}, \tag{3}$$

where $|.|$ are the cardinalities of the prediction and groundtruth sets.

Optimiser Parameters

Name of the optimiser = ADAM

Learning rate = 0.001

Decay rate for 1st moment estimates = 0.9

Decay rate for 2nd moment estimates = 0.999

Cost function = Dice coefficient

During training, the accuracy was evaluated on the validation dataset after each iteration of all the training data through the network. This was repeated until the validation accuracy stopped increasing, and the best performing model was selected for evaluation on the test set. The maximum number of epochs was 100 and 500 steps were set for each epoch. Early stopping with a patience of 50,000 iterations was used as a means of regularisation and to reduce training time.

Training Parameters

Number of epochs = 100

Size of training batch = 16

Size of verification batch = 32

The training was done on two NVIDIA Titan V GPU with 5120 CUDA cores and 12GB of memory each, and took approximately 6 hours. Training error of the segmentation network was also evaluated using Dice, accuracy, sensitivity, specificity, and precision measurements for each of the blood pool, PVs, and MV labels. Table S1 summarises these metrics.

Measurement	Blood Pool	Pulmonary Veins	Mitral Valve
Dice	0.94±0.00	0.67±0.04	0.84±0.06
Accuracy	0.99±0.00	0.99±0.00	0.99±0.00
Sensitivity	0.90±0.01	0.59±0.06	0.85±0.08
Specificity	0.99±0.00	0.99±0.00	0.99±0.00
Precision	0.97±0.01	0.83±0.08	0.85±0.10

Table S1: Evaluation of training error of the CNN network.

Network Model Selection

To select the optimal network architecture, a grid search of hyperparameters was performed using five separate models. Each model was trained and tested on the validation set. The models were permutations of the following hyperparameters: varying depth $m \in 4, 5, 6$, number of feature maps $k \in 32, 64$, and dropout rates $f \in 25, 50, 75$. We chose a depth of 5 and 32 feature maps for the first layer of network. Table S2 summarises Dice scores of segmenting the body of LA using the validation set. Overfitting is a potential issue in larger neural networks due to the large number of trainable parameters. To minimise this issue, dropout rates of 25%, 50%, and 75% were evaluated to find the most effective number of nodes to remove, while still keeping enough nodes for sufficient feature learning. We selected a dropout of 50% as the result of tuning this parameter.

```
# Network's Hyperparameters
number of layers in the net = 5
Number of features in the first layer = 32
Dropout probability = 0.5
```

Model	Network Depth	Feature Maps	No. of Parameters	Dice
A	4	32	1926536	0.86
**B**	**5**	**32**	**7762568**	**0.89**
C	6	32	31100040	0.83
D	4	64	7699208	0.80
E	5	64	31036680	0.85

Table S2: Hyperparameter selection explored in possible models with grid search. Models deeper than 6 with feature maps larger than 64 were too large to fit into GPU memory.

Rigid Registration Implementation

The rigid transform can register objects that are related by rotation and translation by optimising a similarity measure. The source code of registration algorithm can be downloaded from MIRTK GitHub[1]. The set of parameters for use within MIRTK framework can be downloaded from our website[2]. Below is the full list of chosen hyperparameters for the rigid MIRTK registration method:

```
# Registration parameters
Maximum no. of line search iterations = 20
Reuse previous step length = Yes
Strict step length range = Yes
Maximum streak of rejected steps = 5
Transformation model = Rigid
Multi-level transformation = Default
Merge global and local transformation = No
Optimization method = ConjugateGradientDescent
No. of resolution levels = 4
Interpolation mode = Fast linear
Extrapolation mode = Default
Precompute image derivatives = No
Normalize weights of energy terms = Yes
Downsample images with padding = Yes
Crop/pad images = Yes
```

[1]https://github.com/BioMedIA/MIRTK/tree/master/Modules/Registration
[2]https://www.cemrg.co.uk/software/Rigid_MRI.cfg

Crop/pad FFD lattice = Yes
Adaptive surface remeshing = No
Padding value = -1
Resolution [mm] = 1 1 1
Blurring [mm] = -1

Registration parameters for resolution level 1,2,3
Image dissimilarity weight (signed) = 1
Image dissimilarity relative to initial value = Yes
Image dissimilarity approximate gradient = No
Image dissimilarity preconditioning (voxel-wise) = 0
Image dissimilarity preconditioning (node-based) = 0
Image dissimilarity blurring of image gradient = 0
Image dissimilarity blurring of image hessian = 0
Normalize energy gradients (experimental) = No
Energy preconditioning = 0
Maximum no. of iterations = 100
Epsilon = -0.0001
Delta = 1e-12
Maximum no. of line iterations = 20
Maximum streak of rejected steps = 5
Reuse previous step length = Yes
Strict incremental step length range = Yes
Step length rise = 1.1
Step length drop = 0.5
Maximum no. of line search iterations = 20
Strict step length range = Yes
Maximum no. of restarts = 100
Maximum no. of failed restarts = 5
Line search strategy = Adaptive
Blurring [mm] = 0

Registration parameters for resolution level 1
Resolution level = 1
Minimum length of steps = 0.01
Maximum length of steps = 1
No. of bins = 64
Resolution [mm] = 1 1 1

Registration parameters for resolution level 2
Resolution level = 2
Minimum length of steps = 0.02
Maximum length of steps = 2
No. of bins = 64
Resolution [mm] = 2 2 2

Registration parameters for resolution level 3
Resolution level = 3
Minimum length of steps = 0.04
Maximum length of steps = 4
No. of bins = 64
Resolution [mm] = 4 4 4

Registration parameters for resolution level 4

Resolution level = 4

Minimum length of steps = 0.08

Maximum length of steps = 8

No. of bins = 30

Resolution [mm] = 8 8 8

Segmentation Test Results

Permutation Tests

We examined our pipeline efficacy by training on a 70% random selection of scans from all five operators. For further analysis, we also examined training our pipeline on one operator, who had the largest number of scans processed. We then tested this pipeline's performance against independent scans from the same operator and also the 60 scans analysed by the other four operators. The results can be found in tables S3, S4, S5, and S6.

Measurement	Blood Pool	Pulmonary Veins	Mitral Valve
Dice	0.92±0.01	0.65±0.09	0.76±0.08
Accuracy	0.99±0.00	0.99±0.00	0.99±0.00
Sensitivity	0.93±0.01	0.68±0.08	0.88±0.08
Specificity	0.99±0.00	0.99±0.00	0.99±0.00
Precision	0.91±0.03	0.63±0.12	0.69±0.14

Table S3: Average segmentation results obtained from training the CNN network with a random selection of annotations from one operator with the largest number of analysed scans. The testing sets were independent scans made out of annotations from the same operator.

Measurement	Blood Pool	Pulmonary Veins	Mitral Valve
Dice	0.90±0.04	0.66±0.06	0.72±0.08
Accuracy	0.99±0.00	0.99±0.00	0.99±0.00
Sensitivity	0.94±0.03	0.69±0.10	0.76±0.16
Specificity	0.99±0.00	0.99±0.00	0.99±0.00
Precision	0.86±0.06	0.63±0.09	0.72±0.14

Table S4: Average segmentation results obtained from training the CNN network with a random selection of annotations from one operator with the largest number of analysed scans. The testing sets were the 60 scans analysed by the other four operators.

Measurement	IIR 0.97	IIR 1.61	Mean+3.3SD
ICC	0.94	0.99	0.98
PCC	0.94	1.00	0.99
RMSE	2.83	0.03	0.19

Table S5: Fibrosis scores calculated from segmentations generated manually by one operator and automatically by our CNN. ICC is the intra-class correlation coefficients, PCC is the Pearson correlation coefficient, and RMSE is the root mean square error.

Measurement	IIR 0.97	IIR 1.61	Mean+3.3SD
ICC	0.88	0.99	0.98
PCC	0.93	0.99	0.99
RMSE	4.32	0.08	0.38

Table S6: Fibrosis scores calculated from segmentations generated manually by four operators and automatically by our CNN using similar measurement metrics.

Table S3 and S4 represent the results from two separate subsets. Table S3 represents the results from

scans analysed only by one operator in training and testing pools, whereas table S4 represents results from the model that was trained only on scans analysed by one operator and tested on scans analysed by four separate operators, which were completely absent in the training set. Retraining the model on a larger dataset with labelled samples from equally skilled operators will decrease these minor differences, as the available data will be from a more uniform distribution.

Challenge Dataset Tests

Current state-of-the-art deep learning segmentation methods are deep artificial neural networks. We chose the U-Net architecture as recent reviews of cardiac image segmentation methods have confirmed their success in dealing with limited size datasets (4, 5, 6). The proceedings of the 2018 international workshop on statistical atlases and computational models of the heart cover a range of 2D and 3D CNN based methods for segmenting the atria (4). We trained our network on 2D slices of scans, as it was observed that feeding in 3D sets does not have a significant effect on the accuracy of results and requires more processing power. The benchmarking of architectures used in the 2018 Atrial Segmentation Challenge confirmed the lack of significant difference between 2D and 3D models (4, 6).

We additionally tested our CE-MRA based network on 100 LGE-CMR scans from the 2018 Atrial Segmentation Challenge dataset and evaluated its potential limitations on analysing different scans from a different centre. The network without any retraining achieved a Dice score of 0.80 ± 0.05. Our previous work specifically trained on the LGE-CMR scans from the challenge dataset had achieved a Dice score of 0.89 (3). We took these cross-centre evaluations further by processing the scans from the 2013 Left Atrial Segmentation Challenge and performing a direct comparison to Mortazi et al. work (7). By training our network architecture on the 2D planes of scans, we found a LA Dice score of 0.90 ± 0.09, 0.81 ± 0.08, and 0.78 ± 0.07, whereas Mortazi et al. reported Dice scores of 0.90, 0.80, and 0.78 for axial, coronal, and sagittal planes, respectively. The same analysis for PVs showed a Dice score of 0.61 ± 0.09, 0.47 ± 0.08, and 0.40 ± 0.12 for our method, versus their Dice scores of 0.56, 0.47, and 0.39. The combination of LA and PVs resulted in Dice scores of 0.86 ± 0.02, 0.71 ± 0.05, and 0.78 ± 0.07, versus 0.84, 0.69, and 0.73. As Mortazi et al. reported, training three separate networks with identical architecture on each of the planes and combining their outputs result in the Dice scores of 0.95, 0.68, and 0.90 for LA, PVs, and LA and PVs combined. However, this comes at the expense of three times training and running times, in addition to three times of parameters to tune. The segmented PVs from Mortazi et al. work are also merely the continuation of blood pool in the adjacent regions and their algorithm cannot in fact differentiate between the anatomies. Evaluating MV labels were not possible, as this label was not available in any of the datasets.

Previous automatic atrial segmentation work (8, 7, 9, 10) have relied on one expert delineation per subject and do not provide any inter-observer error margin. For example AtriaNet (10), consisted of a dual pathway CNN architecture and was validated on LGE-CMR dataset of 154 patients from the University of Utah. In contrast, we trained our network on 207 scans with labels of blood pool, MV, and PVs. Jia et al. introduces another solution consisting of two successive networks based on the U-Net architecture and a contour loss on a dataset of 100 subjects (9). Their first network was used to locate the target and the second performed single label segmentation from the cropped region of interest. Our experiments showed training one network with sufficient data is able to delineate LA as accurately as a trained operator.

By quantifying the inter-observer variability, we provide an estimate of the error in manual segmentations and an estimate of the degree of accuracy that we could achieve with an automatic segmentation network, given the inherent inaccuracies in the annotation processes, which are subject to operator's interpretation of the blood pool, landmarks defining the MV's plane, and position of PVs ostia. In fact, the network outperformed operators on all the three labels ($p < 0.05$), with the greatest improvement in the PVs and MV (blood pool: 0.91, PVs: 0.61, MV: 0.73). This more accurate automatic segmentation gives rise to improved or equal inter-observer scores between the pipeline and the operators, in comparison to between the operators across all methods for estimating fibrosis.

Studies like (8, 9, 10) segmented LGE-CMR scans. In contrast, we used our clinically validated LGE-CMR interrogation technique (11) and preformed our segmentation on the higher contrast CE-MRA scans. This potentially allowed more accurate segmentations. It is worth noting that the operators were not instructed to segment the PVs fully and were asked to segment enough tissue to localise ostia. Therefore, the suggested proximal PVs manually generated labels varied and a low inter-observer score was seen in the results. Our

pipeline is not influenced by this variance, as the ostia localisation algorithm finds the centre of mass for any quantity of PV segmentation and uses it as a landmark for finding ostia. The automatic clipping tool then removes the unwanted tissue.

Effect of Wall Thickness on Fibrosis Burden

The results of a one way ANOVA in Figure S2 revealed no significant difference ($p = 0.06$) in the global fibrosis burdens calculated by varying the length of the normal projections initiating from the nodes of the atrial surface mesh by considering the significance level as 0.05.

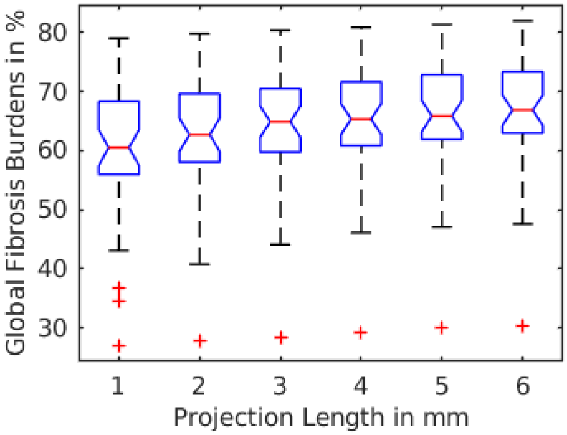

Figure S2: Wall Thickness Analysis: A one way ANOVA test confirms that varying the length of normal projections initiating from the nodes of the mesh does not significantly change the mean of fibrosis burdens.

References

[1] Piccinelli M, Veneziani A, Steinman D A, Remuzzi A, Antiga L. A Framework for Geometric Analysis of Vascular Structures: application to Cerebral Aneurysms *IEEE Transactions on Medical Imaging.* 2009;28:1141 - 1155.

[2] Tobon-Gomez C, Geers AJ, Peters J, Weese J, Pinto K, Karim R, Ammar M, Daoudi A, Margeta J, Sandoval Z, et al. Benchmark for Algorithms Segmenting the Left Atrium From 3D CT and MRI Datasets *IEEE Transactions on Medical Imaging.* 2015;34:1460 - 1473.

[3] Vente C, Veta M, Razeghi O, Niederer S, Pluim J, Rhode K, Karim R. Convolutional Neural Networks for Segmentation of the Left Atrium from Gadolinium-Enhancement MRI Images in *Statistical Atlases and Computational Models of the Heart Atrial Segmentation and LV Quantification Challenges* 2019.

[4] Pop M, Sermesant M, Zhao J, Li S, Mcleod K, Young A, Rhode K, Mansi T. , eds.*Statistical Atlases and Computational Models of the Heart Atrial Segmentation and LV Quantification Challenges*;11395. Springer International Publishing 2019.

[5] Chen C, Qin C, Qiu H, Tarroni G, Duan J, Bai W, Rueckert D. Deep Learning for Cardiac Image Segmentation: A Review *Frontiers in Cardiovascular Medicine.* 2020;7:25.

[6] Zhao J. Towards a Fully Automated MRI-Based Reconstruction of the Left Atrial Cavity 2019.

[7] Mortazi A, Karim R, Rhode KS, Burt JR, Bagci U. CardiacNET: Segmentation of Left Atrium and Proximal Pulmonary Veins from MRI Using Multi-View CNN in *International Conference on Medical Image Computing and Computer Assisted Intervention* 2017.

[8] Tao Q, Ipek EG, Shahzad R, Berendsen FF, Nazarian S, Geest RJ. Fully automatic segmentation of left atrium and pulmonary veins in late gadolinium-enhanced MRI: Towards objective atrial scar assessment *Journal of Magnetic Resonance Imaging.* 2016;44:346 - 354.

[9] Jia S, Despinasse A, Wang Z, Delingette H, Pennec X, Jaïs P, Cochet H, Sermesant M. Automatically Segmenting the Left Atrium from Cardiac Images Using Successive 3D U-Nets and a Contour Loss in *Statistical Atlases and Computational Modeling of the Heart (STACOM) workshop* 2018.

[10] Xiong Z, Fedorov VV, Fu X, Cheng E, Macleod R, Zhao J. Fully Automatic Left Atrium Segmentation From Late Gadolinium Enhanced Magnetic Resonance Imaging Using a Dual Fully Convolutional Neural Network *IEEE Transactions on Medical Imaging.* 2019;38:515 - 524.

[11] Chubb H, Karim R, Mukherjee R, Williams SE, Whitaker J, Harrison J, Niederer SA, Staab W, Gill J, Schaeffter T, et al. A comprehensive multi-index cardiac magnetic resonance-guided assessment of atrial fibrillation substrate prior to ablation: Prediction of long-term outcomes *Journal of Cardiovascular Electrophysiology.* 2019;30:1894 - 1903.
